# Stereotactic Radiosurgery for Intramedullary Spinal Cord Metastases: A Systematic Review and Meta-Analysis

**DOI:** 10.7759/cureus.80025

**Published:** 2025-03-04

**Authors:** Felipe Carvajal, Rafael García, Felipe Rojas, Kita Sallabanda

**Affiliations:** 1 Department of Radiotherapy and Oncology, Hospital Base Valdivia, Valdivia, CHL; 2 Department of Basic Clinical Oncology, Facultad de Medicina, Universidad de Chile, Santiago, CHL; 3 Department of Radiation Oncology, Unidad de Radiocirugía CyberKnife, Hospital Ruber Internacional, Madrid, ESP; 4 Department of Radiosurgery/Neurosurgery, Instituto de Radiocirugía Avanzada (IRCA), Madrid, ESP

**Keywords:** fractionated radiosurgery, intramedullary spinal cord metastases, meningeal neoplasms, spinal neoplasms, stereotactic radiosurgery

## Abstract

Intramedullary spinal cord metastases (ISCM) represent a rare but increasingly diagnosed cancer dissemination. Stereotactic radiosurgery (SRS) and fractionated stereotactic radiosurgery (FSRS) have emerged as a local treatment option in this context over recent years. This systematic review and meta-analysis aim to assess the safety and effectiveness of SRS/FSRS in ISCM. A systematic literature review was conducted following Preferred Reporting Items for Systematic Reviews and Meta-Analyses (PRISMA) guidelines, searching in PubMed/MEDLINE and Google Scholar databases. Studies were selected based on predefined criteria, with bias risk evaluated using Joanna Briggs Institute (JBI) tools. Relevant data were extracted for subsequent meta-analysis. Descriptive statistics and survival analysis using Kaplan-Meier were performed. Ten studies including 60 patients and 77 ISCM treated with SRS/FSRS were selected. The median age was 50 years, with a female predominance (70%). Breast cancer was the most common metastatic origin (41.7%). Kaplan-Meier analysis in 27 patients showed an estimated overall survival (OS) at 12 months of 35.33% (95% CI 0.18-0.53) and at 24 months of 25.98% (95% CI 0.11-0.44), with a median OS of nine months (95% CI 5.2-14). Local control was achieved in 86.3% at the end of follow-up, with favorable neurological control in 69% of patients and no spinal cord toxicity. The findings of this systematic review and meta-analysis suggest that SRS/FSRS appears to be safe and effective in treating ISCM. However, given the low quality of the included studies, these results should be interpreted with caution. Prospective studies are needed to better define the role of SRS/FSRS and evaluate spinal toxicity in this context.

## Introduction and background

Intramedullary spinal cord metastases (ISCM) are a rare form of cancer spread. About 0.1-0.4% of cancer patients will develop ISCM, which is also a rare cause of intramedullary spinal tumors (1-3%) [1]. In recent years, its incidence has increased due to improved diagnostic techniques such as magnetic resonance imaging (MRI) and prolonged survival in metastatic patients because of more effective systemic treatments [2-3]. Historically, ISCM has been considered a late event in the natural course of oncologic disease, associated with poor prognosis [4-7]. The therapeutic management of these lesions is not currently standardized [8]. Local treatment alternatives described in the literature mainly include microsurgical resection and radiotherapy [6-10]. Stereotactic radiosurgery (SRS) and fractionated stereotactic radiosurgery (FSRS) are highly accurate and precise techniques that allow for high-dose treatments in few fractions, achieving excellent results in terms of local control (LC) and sparing of healthy tissues [11-13]. Despite the extensive development of these techniques in recent years, clinical evidence in ISCM remains limited. This systematic review and meta-analysis aim to develop the topic of SRS/FSRS in the context of ISCM to provide an approximate insight into its safety and effectiveness.

## Review

Methods

Literature Search

The search strategy and article selection followed the Preferred Reporting Items for Systematic Reviews and Meta-Analyses (PRISMA) guidelines [14]. The authors conducted a broad search in the PubMed/MEDLINE database (from March 4 to March 8, 2024) and Google Scholar (from March 11 to March 15, 2024), using keywords such as "radiosurgery", "stereotactic radiotherapy", "stereotactic body radiotherapy", "SBRT", "radiotherapy", combined with Boolean operators with "intramedullary metastases". Articles were entered into a Microsoft Excel spreadsheet (Microsoft® Corp., Redmond, WA) for Microsoft 365 MSO Version 2402, and duplicates were removed using the same software.

Article Selection and Bias Risk Assessment

Article selection was performed by two independent reviewers (FC and RG). Excluded articles were cross-evaluated between both reviewers. In case of disagreement, a third reviewer made the final decision (FR). The search strategy began with title and abstract reading. If both met inclusion criteria, the full document was reviewed. Bias risk assessment was conducted using the critical appraisal tools for the risk of bias assessment of included studies developed by the Joanna Briggs Institute (JBI) [15].

Eligibility Criteria

Inclusion criteria considered full articles regarding case reports, case series, retrospective and prospective studies, written in Spanish, English, French, and German languages, published until March 4, 2024, focusing on SRS and FSRS in ISCM, regardless of the number of fractions, and containing information on at least one of the following variables: general clinical data (sex, age, primary histology), general treatment data (total dose, number of fractions, treatment equipment), and outcomes (survival time, assessment of local response, assessment of neurological clinical response). Exclusion criteria included treatment of highly radiosensitive hematologic neoplasms (in which SRS/FSRS is not indicated), use of conventional fractionated radiotherapy (without stereotactic technique), radiosurgery in postoperative context (since there is no macroscopic tumor to target and treat), and articles containing only narrative review information (literature review, systematic review, and meta-analysis without case presentations).

Data Extraction

Data were extracted by two reviewers (FC and RG). A third reviewer (FR) ensured the correctness of the process. Extracted data included the following: author, year of publication, type of article, university or center that published the article and associated country, journal of publication, number of patients and tumors reported per article, age, sex, primary type, presence of concurrent brain metastases, compromised medullary sector, tumor volume, previous radiotherapy in the metastatic sector, equipment used, total dose, number of fractions, prescription isodose, LC, type of local response (complete response, partial response, stable disease, progression), method for defining LC (imaging, clinical), control of neurological symptoms, type of neurological response (stable, improvement, progression), follow-up time, and survival time. All these data were recorded in a Microsoft Excel spreadsheet for Microsoft 365 MSO Version 2402.

Synthesis and Statistical Analysis

Continuous variables were summarized using median and range. Categorical variables were reported as absolute frequencies and percentages. For BED calculation, we used the linear quadratic model. Survival analysis for overall survival (OS) was conducted using the Kaplan Meier method, and presented with 95% Confidence Interval (CI95%). LC was defined as absence of progression, either clinical or on follow-up images. No statistical comparisons were made. Stata version 14.0 was used for these purposes.

Ethical Considerations

This study does not require approval by an ethics committee.

Results

Study Selection

**Figure 1:**
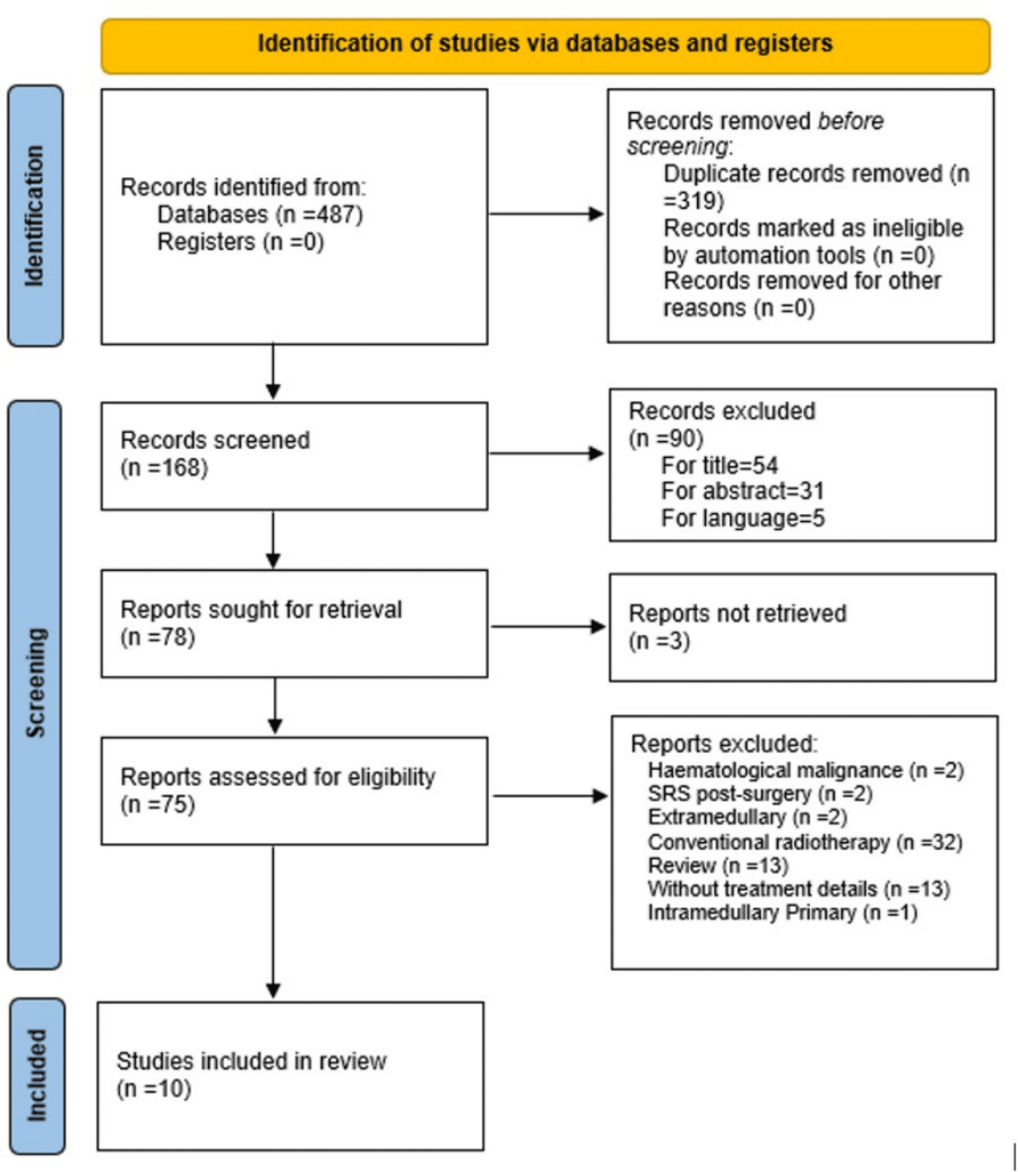
Preferred Reporting Items for Systematic Reviews and Meta-Analyses (PRISMA) 2020 flow diagram

The search strategy resulted in a total of 487 articles. Of these, 319 were removed as duplicates, leaving 168 screened. Among them, 54 were excluded based on title, 31 on abstract, and five due to language. Of the 90 articles previously excluded, 41 were related to bone spine metastases, 18 extramedullary metastases, 12 intramedullary primaries, one study with 2D technique, 13 case reports not related to radiotherapy, and five due to language not considered. Three articles could not be obtained in full, so they were also excluded. Of the 75 full articles evaluated for eligibility, 10 were selected [16-25]. Three of the excluded articles corresponded to case reports of patients treated with FSRS for ISCM but did not meet the predefined criteria. Figure 1 summarizes the study selection process.

Bias Risk Assessment

Seven studies were classified as having medium or high quality, while three of them received a low rating according to JBI instruments (Table 1). Given the low incidence of ISCM, the studies included in this systematic review are mainly case reports and case series. Survival time was extracted from articles, and a Kaplan-Meier analysis was conducted to have an approximate representation of OS, with a high probability of bias. It should be noted that in one of the case series, survival time was published in a graph, so these data were interpolated from the figure for three patients. None of the reports or series conducted a bias analysis.

**Table 1:** Joanna Briggs Institute (JBI) critical appraisal assessment for the risk of bias of included studies

Author	Type of study	Score	Quality
Parikh et al. [17]	Case report	5	High
Shin et al. [18]	Case series	8	High
Dewas et al. [19]	Case report	3	Low
Veeravagu et al. [20]	Case series	8	High
Mori et al. [21]	Case series	0	Low
Garcia et al. [22]	Case report	6	High
Mori et al. [23]	Case report	2	Low
Barrie et al. [24]	Case report	5	Intermediate
Tonneau et al. [25]	Case series	6	High
Ehret et al. [16]	Case series	7	High

Study Characteristics

Of the selected studies, five were case reports (50%), four were single-institution case series (40%), and one was a retrospective multi-institutional series (10%). The included articles were published between 2009 and 2021. Table 2 summarizes the information from the selected articles.

**Table 2:** Description of selected articles aLINAC: Adapted Linear Accelerator; CK: CyberKnife®

Author	Publication year	Journal	University/hospital	Country	Patients	Tumors	Machine
Parikh et al. [17]	2009	Clin Neurol Neurosurg	University of Pittsburgh	USA	1	1	CK
Shin et al. [18]	2009	Neurosurg Focus	Henry Ford Hospital	USA	6	6	aLINAC
Dewas et al. [19]	2011	Revue Neurologique	Université de Lille II	France	1	1	CK
Veeravagu et al. [20]	2012	J Clin Neurosci	Standford University	USA	9	11	CK
Mori et al. [21]	2013	Nagoya Med J	Nagoya University	Japan	2	4	aLINAC
Garcia et al. [22]	2016	Cureus	Imoncology	Spain	1	1	CK
Mori et al. [23]	2016	Cureus	Aichi Medical University	Japan	1	1	aLINAC
Barrie et al. [24]	2020	World Neurosurg	University of Texas Southwestern	USA	1	1	CK
Tonneau et al. [25]	2021	BMC Cancer	Oscar Lambret Center	France	5	5	CK
Ehret et al. [16]	2021	Cancers	Humboldt University, Berlin	Germany	33	46	CK

Data synthesis

Population Characteristics

A total of 60 patients and 77 lesions treated with SRS/FSRS were included, including 31 patients from case reports or case series and 33 patients from a multi-institutional retrospective cohort. The median age was 50 years (11.3-77), with 42 (70%) being female. Concerning the origin of included primary tumors, 41.7% corresponded to breast cancer, 18.3% lung cancer, 8.3% melanoma, 6.7% renal cancer, and 25% other histologies. Brain MRI information was available for 51 patients, with brain metastases present in 38 (75%). Median follow-up duration was eight months (1-72).

Tumor Characteristics

All lesions were detected with MRI. Thirty-five (46%) metastases were located in the cervical spine, 28 (36%) in the thoracic spine, and 14 (18%) in the lumbar spine. Volumetric tumor measurement was available for 73 lesions, with a median volume of 0.75 cc (0.07-15.7 cc). Seven (9%) lesions had received previous radiotherapy at the metastatic site.

Treatment Characteristics

Treatment was performed with CyberKnife® (CK) (Accuray Inc., Sunnyvale, CA) in 66 metastases (86%) and with Adapted Linear Accelerator (aLINAC) 10 Novalis Tx® (Varian Medical Systems, Palo Alto, CA, and Brainlab, Munich, Germany), one Truebeam® (Varian Medical Systems, Palo Alto, CA)) in 11 (14%). The median administered dose was 17 Gy (six to 39) in a median of two fractions (one to 13). Fifty-one (66%) lesions were treated in a single fraction (Table 3).

**Table 3:** Description of treatment schedule Gy: Gray

	Tumors (N)	Median (Gy)	Range (Gy)
One fraction	51	16	6-18
Two fractions	4	20	18-22
Three fractions	8	21	15-27
Four fractions	2	17	14-20
Five fractions	5	25	25
Six to 13 fractions	7	30	16-39

The median administered BED_10_ and BED_3_ were 39 Gy (18.9-57.6) and 86 Gy (30.22-126), respectively. Regarding prescription isodose, the median was 76% (69-100). Table 4 provides a comparison of treatment characteristics between CK and aLINAC equipment.

**Table 4:** Comparison of treatment characteristics between CK and aLINAC aLINAC: Adapted Linear Accelerator; CK: CyberKnife®

	CK (median, range)	aLINAC (median, range)
Total dose	16 Gy (14-36)	16 Gy (10-39)
Fractions	1 (1-6)	2 (1-13)
Prescription isodose	70% (69-97)	90% (80-100)
BED_10_	39.6 (18.9-57.6)	37.5 (20-50.7)
BED_3_	94.6 (30.2-126)	72 (40-101.3)

Oncological Outcomes

**Figure 2:**
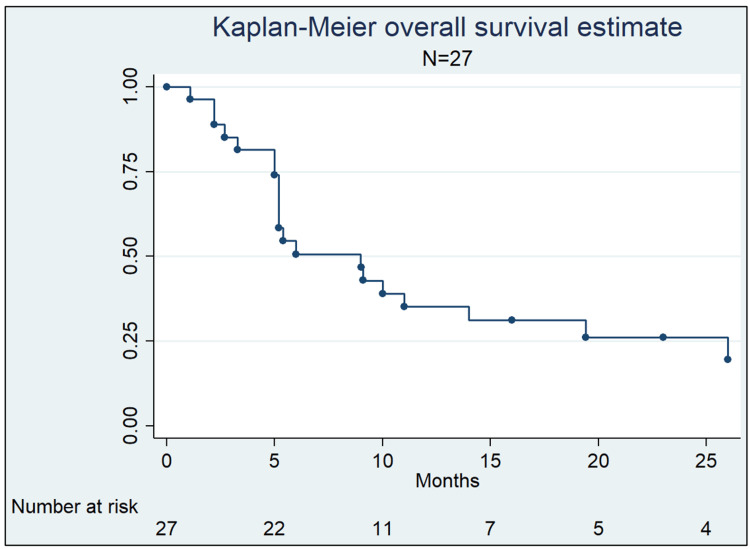
Overall survival in 27 patients treated with stereotactic radiosurgery (SRS)/fractionated stereotactic radiosurgery (FSRS) for intramedullary spinal cord metastases (ISCM)

Based on published data from 27 patients not included in the study by Ehret et al. [16], 12- and 24-month actuarial OS was 35.33% (95% CI 0.18-0.53) and 25.98% (95% CI 0.11-0.44) respectively (Figure 2, Table 5), with a median survival of nine months (95% CI 5.2-14). LC determination was possible in 74 lesions, with an LC rate of 86.3% (95% CI 0.761-0.925) at the end of the follow-up period. This evaluation was conducted in 64 patients (83%) with imaging, seven (9%) clinically, and in six (8%) without information of the method. Considering only lesions evaluated through imaging, the LC rate was 84.3% (95% CI 0.729-0.915).

**Table 5:** Overall survival in 27 patients treated with stereotactic radiosurgery (SRS)/fractionated stereotactic radiosurgery (FSRS) for intramedullary spinal cord metastases (ISCM)

Follow-up (months)	Survival (%)	95% CI
One	96.30	0.7649-0.9947
Three	81.48	0.6109-0.9184
Six	51.03	0.3096-0.6797
12	35.33	0.1798-0.5321
16	31.17	0.1481-0.4909
24	25.98	0.1077-0.4425

Table 6 is a comparison of the characteristics and results of 27 patients included in the survival analysis of this review and the 33 patients from the study published by Ehret et al. [16].

**Table 6:** Characteristics of the Ehret et al. series [16] and the 27 patients included in the survival analysis in this review Gy: Gray; mOS: median overall survival; NR: not reported; OS: overall survival

Characteristic	This review (N = 27)	Ehret et al. (N = 33)
Age (median, years)	62	49.2
Sex (female)	67%	73%
Primary tumor		
Breast	33.3%	48%
Lung	25.9%	12%
Melanoma	7.4%	9%
Renal cell	14.8%	NR
Other	18.5%	31%
Total dose Gy (median, range)	21 (10-39)	16.1 (6-24)
Fractions (median, range)	3 (1-13)	1.1 (1-3)
mOS (months)	9	11.7
One-year OS	35.3%	47.5%

Functional Outcomes

Neurological response was not assessed using a standard scale across different publications. Regarding reported information, neurological evaluation was not recorded for 11 patients (18%), there was neurological improvement in 19 patients (32%), stability in 22 (37%), and deterioration in eight (13%). Overall, good neurological control was observed in 69% of patients treated with FSRS included in this review (95% CI 0.71-0.92). There were no reports of spinal cord toxicity (95% CI 0.00-0.05).

Discussion

ISCM represents an infrequent clinical context, with scarce evidence published. Consequently, there are no guidelines for the best treatment selection in different clinical settings [8]. This systematic review and meta-analysis, to our knowledge, is the first exclusively focused on SRS/FSRS for intramedullary metastases. Historically, ISCM was associated with poor prognosis, with a median OS of around two to four months [2,4-7].

The main objectives in the treatment of ISCM are to preserve neurological function, achieve LC, and avoid treatment-associated toxicities. Ehret et al. [16] conducted a retrospective multi-institutional study in Germany, recruiting a total of 33 patients with 46 ISCM treated with SRS/FSRS. The median survival was 11.7 months, with a one- and two-year survival of 47.5% and 31.6%, respectively. In the survival analysis conducted with 27 patients in our review, the estimated median survival was nine months and the two-year survival was 26%. Both results show a favorable prognosis compared to historical publications and are similar to those found in major phase III studies of brain metastases (7.5-10.9 months), clinical setting in which intensive local management with surgery and/or SRS is a standard [26-29].

The better median survival observed in Ehret et al.'s study [16] may be related to a higher inclusion of patients with breast cancer (48% vs. 33% in our analysis), whose prognosis is favorable compared to, for example, lung cancer, whose proportion was comparatively lower (12% vs. 26%, respectively). Additionally, as discussed in Ehret et al.'s study [16], the patient inclusion period (since 2005) may also impact the availability of systemic treatments. The fact that more than 25% of patients are alive at two years reinforces the need to apply highly effective treatments to prevent neurological progression and early paralysis. The results of this review show good clinical neurological control in 69% of patients during the follow-up period, with only eight (13%) of them experiencing deterioration. These results of LC and neurological symptom control support considering SRS/FSRS as a suitable option in the treatment of patients with ISCM.

Surgery is another treatment option described in the literature, mainly considered when histological sampling is required to confirm malignancy, in patients with a prolonged life expectancy (solitary ISCM, good performance status, long disease-free interval), and in cases with accelerated neurological deterioration [8,30,31]. Sutter et al. described three phases to consider within the management of ISCM. The first phase, from the initial symptoms until motor paresis or sphincter involvement, the second until paraplegia, and the third from paraplegia until death. According to the author, surgery would be mainly indicated during the second phase, especially for tumors with lower radiosensitivity [32].

Gazzeri et al. reported the outcomes of 30 patients operated on for ISCM [33]. The surgical objective was macroscopic tumor resection (gross total resection (GTR)), but was achieved in only 17 patients (56.7%). Neurological functionality, defined based on the modified McCormick scale scores I and II, was 33.3% preoperatively and 46.6% postoperatively. They obtained a median survival of 11.1 months in patients with subtotal resection (STR) and 11.3 months with GTR; however, 36.6% of patients experienced surgery-related adverse effects. In another publication, Gazzaeri et al. developed a prognostic score for operated patients based on clinical information from 33 patients, with a score of 0-10. Variables included age, general status, type of primary tumor, neurological symptoms, and the presence of extramedullary disease [30]. Patients with scores of 0-3, 4-5, and 6-10 had a median survival of three months, 7.6 months, and 14.8 months, respectively, a difference that was statistically significant (p = 0.001). They concluded that patients who would benefit most from surgery are those with scores of 6-10. Although there is no consensus postoperative treatment, most patients in surgical series received adjuvant radiotherapy and/or chemotherapy [34].

The main disadvantage of surgery is the complex and invasive nature of the procedure and its association with potential post-procedure complications that could affect the quality of life in metastatic patients [35].

Conventional radiotherapy allows the application of adequate palliative dose for intramedullary lesions [6,7,36], but in a greater number of fractions, increasing total treatment time and the number of hospital visits, important points to consider in palliative management [37]. Additionally, due to its poorer technical quality indices (gradient and conformation), dose administered via conventional radiotherapy affects a significantly larger volume of spinal cord, which in animal models has been associated with increased toxicity [38,39]. Finally, there are tumors with a low α/β ratio that benefit from the extreme hypofractionation allowed by stereotactic techniques, resulting in a higher biologically effective dose and better LC. In fact, 42% of the cases included in this review corresponded to patients with breast cancer, with an estimated α/β ratio of 3.7 [40]. The difference in BED_3_ between CK and aLINAC equipment (89.92 Gy_3_ vs. 69.66 Gy_3_) observed in our review demonstrates the importance of appropriately selecting the technological platform that allows the greatest possible hypofractionation in tumors with low α/β ratios.

Despite the benefits of SRS/FSRS, there are patients with high symptomatic burden who cannot tolerate the required positioning for an extended period, as occurs in some patients with vertebral metastases [41]. These cases should be considered for conventional radiotherapy treatment. Schiff and O’Neill retrospectively reviewed 40 patients with intramedullary metastases, of whom 35 received conventional radiotherapy, with a dose ranging from 16.3 to 45.2 Gy (mean of 30 Gy) in five to 25 fractions (mean of 10). The median survival in the complete series was three months, with six patients (15%) surviving more than one year. Patients with breast cancer had a median survival of 13 months. The median survival in patients who received radiotherapy was four months versus two months in those who did not receive it. At the last follow-up, 90% of patients maintained the same neurological functional status as at hospital discharge [7].

Although craniospinal irradiation (CSI) could be considered a reasonable option for patients with leptomeningeal dissemination, CSI is not commonly used in this context due to associated risks, including bone marrow suppression (up to 37% of cases), enteritis, mucositis, and the probable concomitance with systemic therapies that are associated with increased toxicity. It has also been reported that nearly half of these patients fail to complete the craniospinal treatment. The European Society for Medical Oncology (ESMO) considers radiotherapy a useful therapeutic tool as it achieves rapid improvement in neurological symptoms. However, since it has not shown any improvement in survival, it does not recommend it as a standard first-line treatment for recently diagnosed patients with asymptomatic leptomeningeal involvement. In cases where it is performed, they recommend the use of focal radiotherapy, either hypofractionated or SRS/FSRS, especially in the management of symptomatic brain and/or spinal nodular disease, primarily in those with better clinical prognostic factors [42]. The German Society for Radiation Oncology (DEGRO) recommends that in leptomeningeal carcinomatosis, both local (whole-brain) radiotherapy and local spinal radiotherapy should be considered alongside systemic therapy, and only in patients with good clinical condition and limited or stable extra-CNS disease should CSI be considered [43]. This is also consistent with the recommendations of European Association of Neuro-Oncology (EANO) 2024 [44].

The main risk associated with radiosurgery treatment in intramedullary tumors is radiation-induced myelopathy, whose symptoms can range from motor, sensory, and sphincter alterations to paraplegia/quadriplegia and loss of autonomic function [45]. In the present review, no spinal toxicity events were observed; however, it should be considered that myelopathy is a late adverse effect, and considering the low median survival and follow-up of the patients included in this review, its actual incidence may not have been demonstrated. Additionally, the maximum dose in the spinal cord was not recorded in the selected articles, so we cannot draw specific conclusions about it.

Dose tolerance limits for the spinal cord have been systematically studied by Sahgal et al. [45-47], standardizing limits that maintain the risk of myelopathy below 1-5%. These dose constraints were analyzed, accepted, and recommended by the Hypofractionation Treatment Effects in the Clinic (HYTEC) group [45], including based on the studies of Katsoulakis et al. [48], a maximum dose of up to 14 Gy for a single fraction. 

Daly et al. [49] evaluated spinal cord tolerance to high doses in a retrospective series of 19 patients with 27 spinal hemangioblastomas treated with SRS/FSRS using CK. The median maximum dose in a single fraction on the spinal cord was 22.7 Gy, with spot doses of up to 30.9 Gy, without recording spinal toxicity G2+. The authors partly explain this observation based on the small irradiated volume with a high dose gradient, consistent with published animal models [38,39].

Despite the experience in hemangioblastoma, the current recommendation is to prioritize the spinal cord dose limit over tumor coverage, since there is not enough information about incidence of myelopathy with higher doses [50]. It is essential to develop prospective clinical studies to evaluate this topic specifically in the context of ISCM.

**Figure 3:**
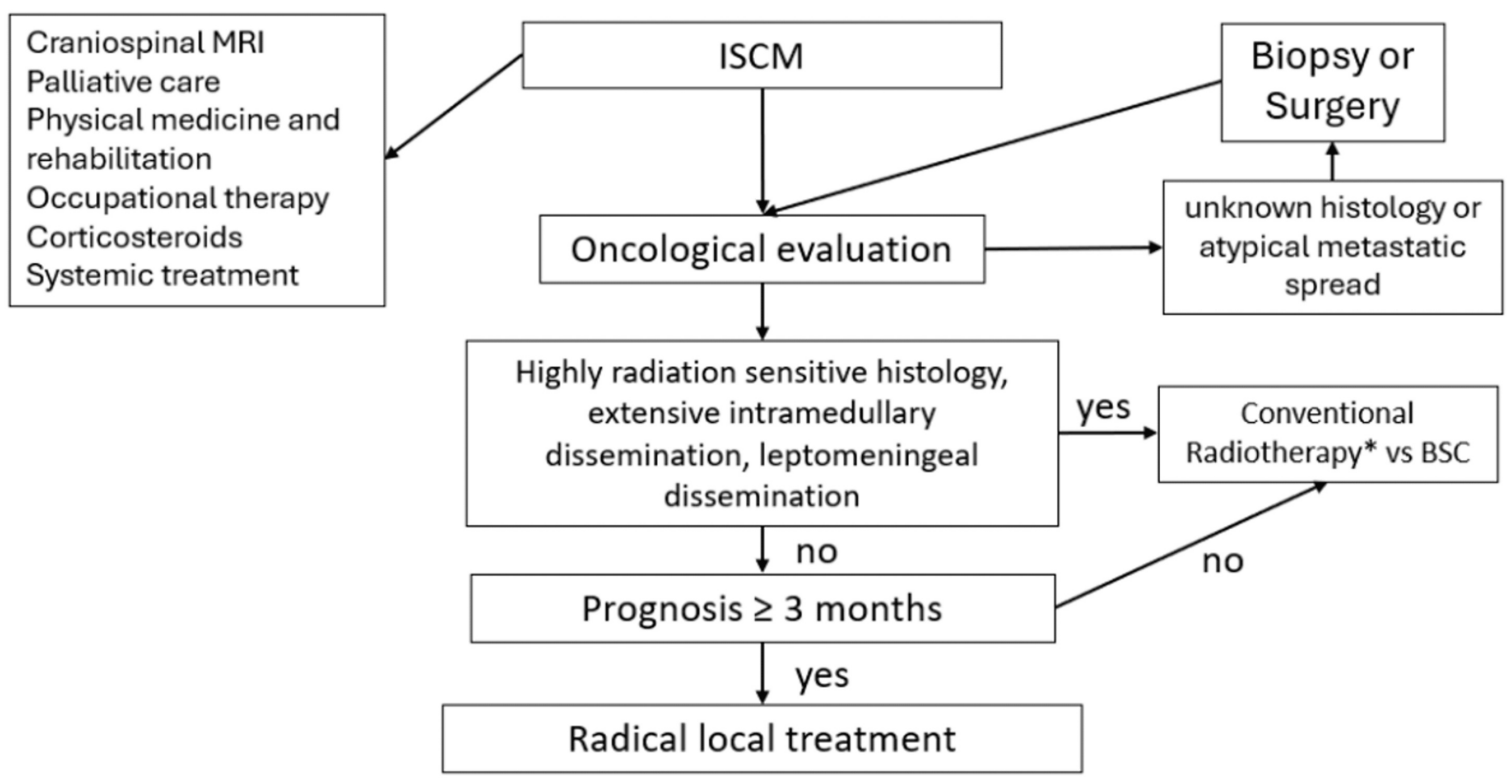
Initial management and patient selection algorithm for radical local treatment in ISCM BSC: best supportive care; ISCM: intramedullary spinal cord metastases; MRI: magnetic resonance imaging *Includes craniospinal radiotherapy

**Figure 4:**
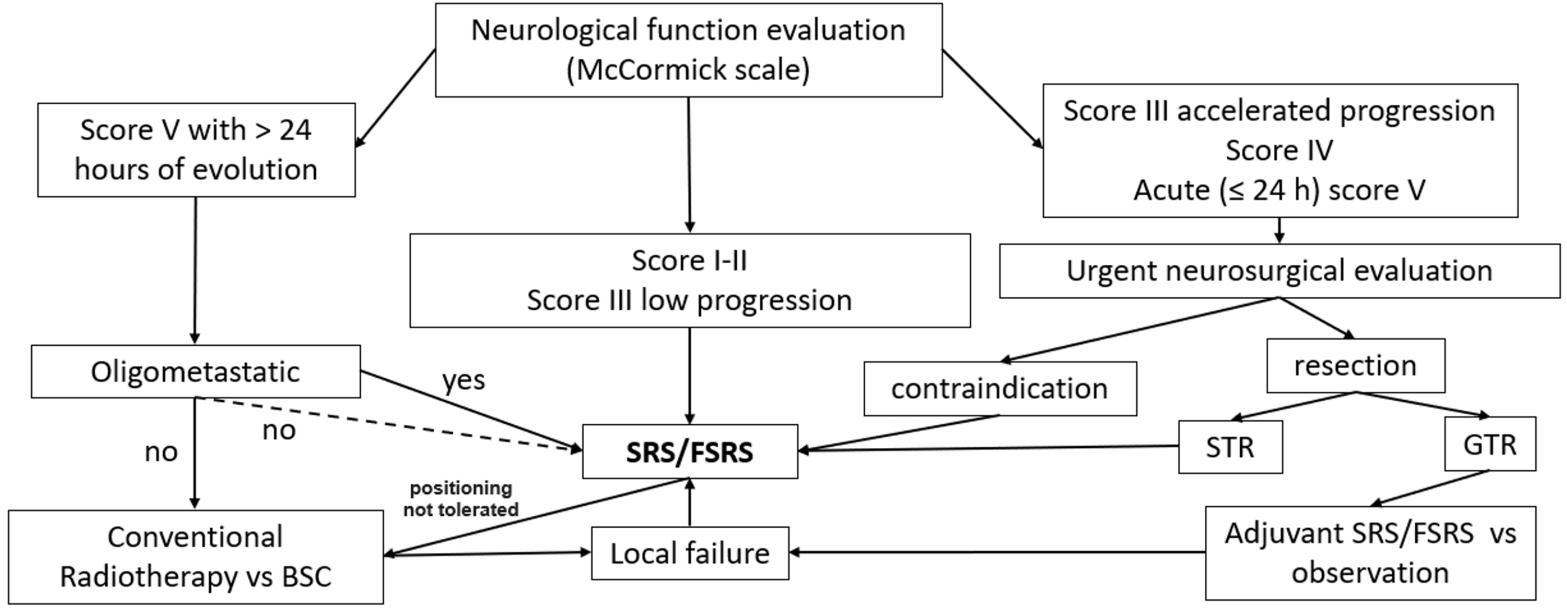
Selection of patients with ISCM for treatment with SRS/FSRS BSC: best supportive care; FSRS: fractionated stereotactic radiosurgery; GTR: gross total resection; ISCM: intramedullary spinal cord metastases; SRS: stereotactic radiosurgery; STR: subtotal resection

Finally, all patients should undergo assessment for symptomatic management by the palliative care unit, physical therapy and rehabilitation unit, and medical oncology to indicate the best available systemic treatment according to histology type and patient status; multimodal management has shown benefits in patients with intramedullary metastases [9]. Figure 3 and Figure 4 show a proposed scheme of clinical decisions, focusing on the use of SRS/FSRS.

This review has several limitations. First, the data source (mainly case reports and case series) represents the lowest level of available evidence. The lack of bias assessment, confounding factors evaluation, detailed information regarding the dosimetric plan, and standardization of management and follow-up among the articles imply that the results should be considered with caution. On the other hand, there could be reporting and publication bias of cases, which would imply better results in the analysis than what actually occurred in the population. Lastly, the explosive development of systemic treatments in recent years has dramatically improved OS in metastatic patients, an event that by the publication date of the included articles may not be fully reflected in this review.

## Conclusions

Clinical evidence related to SRS/FSRS in ISCM is limited, of low quality, and mainly consists of case reports and case series. This systematic review shows that this non-invasive technique, applied with the appropriate technology, appears to be safe and effective, achieving high rates of LC, neurological preservation, and low risk of spinal cord toxicity. It is important to develop prospective protocols and clinical trials that allow delineating the real role of SRS/FSRS and spinal cord toxicity in the context of ISCM.

## References

[1] Kalayci M, Cağavi F, Gül S, Yenidünya S, Açikgöz B (2004). Intramedullary spinal cord metastases: diagnosis and treatment - an illustrated review. Acta Neurochir (Wien).

[2] Lv J, Liu B, Quan X, Li C, Dong L, Liu M (2019). Intramedullary spinal cord metastasis in malignancies: an institutional analysis and review. Onco Targets Ther.

[3] Payer S, Mende KC, Westphal M, Eicker SO (2015). Intramedullary spinal cord metastases: an increasingly common diagnosis. Neurosurg Focus.

[4] Edelson RN, Deck MD, Posner JB (1972). Intramedullary spinal cord metastases. Clinical and radiographic findings in nine cases. Neurology.

[5] Bagley CA, Gokaslan ZL (2004). Cauda equina syndrome caused by primary and metastatic neoplasms. Neurosurg Focus.

[6] Lee SS, Kim MK, Sym SJ, Kim SW, Kim WK, Kim SB, Ahn JH (2007). Intramedullary spinal cord metastases: a single-institution experience. J Neurooncol.

[7] Schiff D, O'Neill BP (1996). Intramedullary spinal cord metastases: clinical features and treatment outcome. Neurology.

[8] Sung WS, Sung MJ, Chan JH (2013). Intramedullary spinal cord metastases: a 20-year institutional experience with a comprehensive literature review. World Neurosurg.

[9] Saeed H, Patel R, Thakkar J, Hamoodi L, Chen L, Villano JL (2017). Multimodality therapy improves survival in intramedullary spinal cord metastasis of lung primary. Hematol Oncol Stem Cell Ther.

[10] Hernández-Durán S, Hanft S, Komotar RJ, Manzano GR (2016). The role of stereotactic radiosurgery in the treatment of intramedullary spinal cord neoplasms: a systematic literature review. Neurosurg Rev.

[11] Koong AC, Tepper JE (2017). Introduction. Semin Radiat Oncol.

[12] Timmerman RD, Forster KM, Chinsoo Cho L (2005). Extracranial stereotactic radiation delivery. Semin Radiat Oncol.

[13] Papiez L, Timmerman R, DesRosiers C, Randall M (2003). Extracranial stereotactic radioablation: physical principles. Acta Oncol.

[14] Page MJ, Moher D, Bossuyt PM (2021). PRISMA 2020 explanation and elaboration: updated guidance and exemplars for reporting systematic reviews. BMJ.

[15] Munn Z, Barker TH, Moola S (2020). Methodological quality of case series studies: an introduction to the JBI critical appraisal tool. JBI Evid Synth.

[16] Ehret F, Senger C, Kufeld M (2021). Image-guided robotic radiosurgery for the management of intramedullary spinal cord metastases-a multicenter experience. Cancers (Basel).

[17] Parikh S, Heron DE (2009). Fractionated radiosurgical management of intramedullary spinal cord metastasis: a case report and review of the literature. Clin Neurol Neurosurg.

[18] Shin DA, Huh R, Chung SS, Rock J, Ryu S (2009). Stereotactic spine radiosurgery for intradural and intramedullary metastasis. Neurosurg Focus.

[19] Dewas S, Le Rhun E, Duhem R, Dansin E, Prevost B, Lartigau E (2011). Solitary intramedullary metastasis from malignant pleural mesothelioma treated with CyberKnife®: a case report. Rev Neurol (Paris).

[20] Veeravagu A, Lieberson RE, Mener A (2012). CyberKnife stereotactic radiosurgery for the treatment of intramedullary spinal cord metastases. J Clin Neurosci.

[21] Mori Y, Hashizume C, Shibamoto Y, Kobayashi T, Nakazawa H, Hagiwara M, Tsuwawa T (2013). Stereotactic radiotherapy for spinal intradural metastases developing within or adjacent to the previous irradiation field - report of three cases -. Nagoya J Med Sci.

[22] Garcia R, Sallabanda K, Santa-Olalla I (2016). Robotic radiosurgery for the treatment of intramedullary spinal cord metastases: a case report and literature review. Cureus.

[23] Mori Y, Kawamura T, Ohshima Y, Takeuchi A, Mori T, Ishiguchi T (2016). Stereotactic radiotherapy for cervical spinal intramedullary metastasis and multiple brain metastases: a case report. Cureus.

[24] Barrie U, Elguindy M, Pernik M (2020). Intramedullary spinal metastatic renal cell carcinoma: systematic review of disease presentation, treatment, and prognosis with case illustration. World Neurosurg.

[25] Tonneau M, Mouttet-Audouard R, Le Tinier F, Mirabel X, Pasquier D (2021). Stereotactic body radiotherapy for intramedullary metastases: a retrospective series at the Oscar Lambret center and a systematic review. BMC Cancer.

[26] Aoyama H, Shirato H, Tago M (2006). Stereotactic radiosurgery plus whole-brain radiation therapy vs stereotactic radiosurgery alone for treatment of brain metastases: a randomized controlled trial. JAMA.

[27] Brown PD, Jaeckle K, Ballman KV (2016). Effect of radiosurgery alone vs radiosurgery with whole brain radiation therapy on cognitive function in patients with 1 to 3 brain metastases: a randomized clinical trial. JAMA.

[28] Chang EL, Wefel JS, Hess KR (2009). Neurocognition in patients with brain metastases treated with radiosurgery or radiosurgery plus whole-brain irradiation: a randomised controlled trial. Lancet Oncol.

[29] Kocher M, Soffietti R, Abacioglu U (2011). Adjuvant whole-brain radiotherapy versus observation after radiosurgery or surgical resection of one to three cerebral metastases: results of the EORTC 22952-26001 study. J Clin Oncol.

[30] Gazzeri R, Telera S, Galarza M, Sperduti I, Alfieri A (2023). Prognostic scoring system for surgical treatment of intramedullary spinal cord metastases. J Clin Neurosci.

[31] Goyal A, Yolcu Y, Kerezoudis P, Alvi MA, Krauss WE, Bydon M (2019). Intramedullary spinal cord metastases: an institutional review of survival and outcomes. J Neurooncol.

[32] Friehs GM, Legat J, Zheng Z, Pendl G, Noren GC (1998). Outcomes in patients treated with gamma knife radiosurgery for brain metastases from malignant melanoma. Neurosurg Focus.

[33] Gazzeri R, Telera S, Galarza M, Callovini GM, Isabella S, Alfieri A (2021). Surgical treatment of intramedullary spinal cord metastases: functional outcome and complications-a multicenter study. Neurosurg Rev.

[34] Kritikos M, Vivanco-Suarez J, Teferi N (2024). Survival and neurological outcomes following management of intramedullary spinal metastasis patients: a case series with comprehensive review of the literature. Neurosurg Rev.

[35] Mut M, Schiff D, Shaffrey ME (2005). Metastasis to nervous system: spinal epidural and intramedullary metastases. J Neurooncol.

[36] Conill C, Marruecos J, Verger E (2007). Clinical outcome in patients with intramedullary spinal cord metastases from lung cancer. Clin Transl Oncol.

[37] Lutz ST, Chow EL, Hartsell WF, Konski AA (2007). A review of hypofractionated palliative radiotherapy. Cancer.

[38] Bijl HP, van Luijk P, Coppes RP, Schippers JM, Konings AWT, van der Kogel AJ (2002). Dose-volume effects in the rat cervical spinal cord after proton irradiation. Int J Radiat Oncol Biol Phys.

[39] Hopewell JW, Morris AD, Dixon-Brown A (1987). The influence of field size on the late tolerance of the rat spinal cord to single doses of X rays. Br J Radiol.

[40] Murray Brunt A, Haviland JS, Wheatley DA (2020). Hypofractionated breast radiotherapy for 1 week versus 3 weeks (FAST-Forward): 5-year efficacy and late normal tissue effects results from a multicentre, non-inferiority, randomised, phase 3 trial. Lancet.

[41] Pielkenrood BJ, van der Velden JM, van der Linden YM (2021). Pain response after stereotactic body radiation therapy versus conventional radiation therapy in patients with bone metastases-a phase 2 randomized controlled trial within a prospective cohort. Int J Radiat Oncol Biol Phys.

[42] Le Rhun E, Weller M, van den Bent M (2023). Leptomeningeal metastasis from solid tumours: EANO-ESMO Clinical Practice Guideline for diagnosis, treatment and follow-up. ESMO Open.

[43] Borm KJ, Behzadi ST, Hörner-Rieber J (2024). DEGRO guideline for personalized radiotherapy of brain metastases and leptomeningeal carcinomatosis in patients with breast cancer. Strahlenther Onkol.

[44] Wilcox JA, Chukwueke UN, Ahn MJ (2024). Leptomeningeal metastases from solid tumors: a Society for Neuro-Oncology and American Society of Clinical Oncology consensus review on clinical management and future directions. Neuro Oncol.

[45] Sahgal A, Chang JH, Ma L (2021). Spinal cord dose tolerance to stereotactic body radiation therapy. Int J Radiat Oncol Biol Phys.

[46] Sahgal A, Ma L, Gibbs I (2010). Spinal cord tolerance for stereotactic body radiotherapy. Int J Radiat Oncol Biol Phys.

[47] Sahgal A, Weinberg V, Ma L (2013). Probabilities of radiation myelopathy specific to stereotactic body radiation therapy to guide safe practice. Int J Radiat Oncol Biol Phys.

[48] Katsoulakis E, Jackson A, Cox B, Lovelock M, Yamada Y (2017). A detailed dosimetric analysis of spinal cord tolerance in high-dose spine radiosurgery. Int J Radiat Oncol Biol Phys.

[49] Daly ME, Choi CY, Gibbs IC, Adler JR Jr, Chang SD, Lieberson RE, Soltys SG (2011). Tolerance of the spinal cord to stereotactic radiosurgery: insights from hemangioblastomas. Int J Radiat Oncol Biol Phys.

[50] Sanford NN, Timmerman RD (2024). Optimizing risk vs. reward in the era of ablative radiotherapy through calculated useful trauma (CUT). Int J Radiat Oncol Biol Phys.

